# MiR-34a-5p suppresses cutaneous squamous cell carcinoma progression by targeting SIRT6

**DOI:** 10.1007/s00403-024-03106-w

**Published:** 2024-05-31

**Authors:** Sai Chen, Muxing Yuan, Hongxia Chen, Tong Wu, Tianqi Wu, Dongmei Zhang, Xu Miao, Jian Shi

**Affiliations:** 1 Affiliated Hospital 2 of Nantong University, Nantong, China; 2 Nantong University, Nantong, China; 3 Medical Research Center, Affiliated Hospital 2 of Nantong University, Nantong, China

**Keywords:** Cutaneous squamous cell carcinoma, Biological behavior, MiR-34a-5p, SIRT6

## Abstract

Cutaneous squamous cell carcinoma (cSCC) is a malignant tumor originating from epidermal or appendageal keratinocytes, with a rising incidence in recent years. Understanding the molecular mechanism driving its development is crucial. This study aims to investigate whether miR-34a-5p is involved in the pathogenesis of cSCC by targeting Sirtuin 6 (SIRT6).The expression levels of miR-34a-5p and SIRT6 were determined in 15 cSCC tissue specimens, 15 normal tissue specimens and cultured cells via real-time polymerase chain reaction (RT-qPCR). Pearson’s correlation analysis was conducted to evaluate the relationship between miR-34a-5p and SIRT6 expression levels in cSCC tissues. A431 and SCL-1 cells were transfected with miR-34a-5p mimic, negative control or miR-34a-5p mimic together with recombinant plasmids containing SIRT6 gene. Cell counting kit-8, clone formation assay, wound healing assay, and flow cytometry were employed to assess the effects of these transfections on proliferation, migration, and apoptosis, respectively. The interaction between miR-34a-5p and SIRT6 was characterized using a dual-luciferase reporter assay.MiR-34a-5p expression was down-regulated in cSCC tissues significantly, while the SIRT6 expression was the opposite. A negative correlation was observed between the expression of miR-34a-5p and SIRT6 in cSCC tissues. Furthermore, overexpression of miR-34a-5p led to a significant reduction in the proliferation and migration abilities of A431 and SCL-1 cells, accompanied by an increase in apoptosis levels and a decrease in SIRT6 expression levels. MiR-34a-5p was identified as a direct target of SIRT6. Importantly, overexpression of SIRT6 effectively counteracted the inhibitory effect mediated by miR-34a-5p in cSCC cells.Our findings suggest that miR-34a-5p functions as a tumor suppressor in cSCC cells by targeting SIRT6.

## Introduction

Cutaneous squamous cell carcinoma (cSCC) is the second most common type of skin cancer, accounting for approximately 10 to 20% of all skin cancer-related deaths [1]. Despite advancements in diagnosis and treatment, the 10-year survival rate for cSCC patients diminishes to less than 20% upon the occurrence of local lymph node metastasis or distant metastasis [2, 3]. However, the molecular mechanism underlying the progression of cSCC has not been fully elucidated.

In previous studies, our research group found that SIRT6 protein is highly expressed in CSCC, and the high expression of SIRT6 promotes the proliferation, migration and invasion of CSCC cells and inhibits their apoptosis, which may play a role as an oncogene. In order to further explore the relevant signaling pathway of SIRT6 in CSCC, We looked for upstream micrornas. MicroRNAs (miRNAs) are small endogenous non-coding RNAs that play crucial roles in various biological and pathological processes as post-transcriptional regulators of gene expression [4, 5]. They are involved in the pathogenesis of different types of cancer, serving as either tumor promoters or inhibitors [6]. Aberrant expression of miR-34a-5p has been reported in both lung and liver cancer, where it demonstrates inhibitory effects on tumor cell growth [7, 8]. However, the function role of miR-34a-5p in the development of cSCC remain poorly understood. Computational target gene prediction indicates Sirtuin 6 (SIRT6) as a potential target of miR-34a-5p, yet its involvement in the regulatory network of miR-34a-5p in cSCC remain uncertain.

In this study, we detected the expression levels of miR-34a-5p and SIRT6 in cSCC cells and tissues. Their functional roles in the progression of cSCC were investigated as well. Our findings aim to provide new insights for the diagnosis and treatment of cSCC.

## Materials and methods

### Materials

#### Tissue sources

A total of 15 cSCC patients were enrolled in this study, including 9 males and 6 females, with ages ranging from 61 to 87 years and a mean age of (77 ± 7.23) years. All of them received treatment at our hospital between December 2021 and December 2022. Surgically resected cSCC tissues were collected, and all patients were pathologically confirmed to have cSCC without receiving antitumor therapy prior to surgery. Fifteen samples of normal skin tissue obtained from cosmetic surgery were used as controls. No significant differences were observed in demographic characteristics between the two groups. Written consent was obtained from all participants, and this study was approved by the ethics committee of our hospital.

#### Cells and reagents

Human cSCC cell lines A431 (purchased from Zhong Qiao Xin Zhou, Shanghai) and SCL-1 (purchased from Enzyme Research Biotech, Shanghai) were used. Skim milk, chloroform, and isopropanol were obtained from Shanghai Sangon Biotech Co., Ltd. Primary antibodies against Cleaved-caspase3, SIRT6, and GAPDH, as well as secondary anti-mouse immunoglobulin IgG, were purchased from Abcam (USA). Fetal bovine serum and DMEM high-glucose medium were acquired from Gibco (USA). The BCA assay kit was sourced from Beyotime Biotechnology Co., Ltd. Polyvinylidene fluoride (PVDF) membranes were obtained from Millipore (USA). Trizol reagent, RNA prep pure Tissue Kit, PrimeScript RT synthesis kit, and SYBR Green qRT-PCR Master Mix kit were all purchased from Thermo Fisher Scientific (USA). The CCK-8 kit was sourced from Proteintech (Wuhan). All primers were synthesized by Shanghai Sangon Biotech Co., Ltd.

### Methods

#### RT-qPCR detection

RNA isolation and quantification were performed using standard protocols. Total RNA was extracted from the tissues using Trizol reagent, followed by chloroform/isopropanol extraction to separate the RNA precipitate. The precipitate was further purified using ethanol precipitation, and the resulting total RNA was dissolved in 20 μL of diethylpyrocarbonate (DEPC)-treated water. One microgram of RNA was reverse-transcribed into cDNA using the PrimeScript RT synthesis kit. Quantitative real-time PCR (qRT-PCR) was then conducted using the SYBR Green qRT-PCR Master Mix kit, with U6 serving as the endogenous control for normalization. Primer sequences are provided in Table 1.

**Table 1:** Primer sequences for real-time fluorescent quantitative PCR

Gene	Primer sequence (5′ to 3′)
miR-34a-5p	F: 5′-CGCGTGGCAGTGTCTTAGCT-3′
	R: 5′-AGTGCAGGGTCCGAGGTATT-3′
	Stem-loop RT: 5′-GTCGTATCCAGTGCAGGGTCCGAGGTATTCGC ACTGGATACGACACAACC-3′
SIRT6	F: 5′-TGGCAGTCTTCCAGTGTGGTGT-3′
	R: 5′-CGCTCTCAAAGGTGGTGTCGAA-3′
U6	F:5′-ATTGGAACGATACAGAGAAGATT-3′
	R: 5′-GGAACGCTTCACGAATTTG-3′

#### Cell culture and grouping

A431, SCL-1, and HaCaT cells were cultured in DMEM medium containing 10% fetal bovine serum (FBS) and incubated at 37 °C in a humidified atmosphere of 5% CO_2_. Lentiviral miR-mimic, control miR-NC, and OE-SIRT6 were provided by Youxi Weinan Biotech Co., Ltd. (Sanming, China). The lentiviruses were packaged using a plasmid system. Following infection of A431 and SCL-1 cells with the lentiviruses for 24 h, stable transduced cell lines were selected using puromycin (0.8 μg/ml for A431 cells and 0.4 μg/ml for SCL-1 cells). The experimental groups were designed as follows: control group (miR-NC), experimental group (miR-mimic), and OE-SIRT6 + miR-mimic group.

#### Cell viability assay using CCK-8

Cells were seeded at a density of 2.0 × 10^3^ cells/100 μl in 96-well plates, with four replicates per group. They were incubated at 37 °C in a humidified atmosphere of 5% CO2 with saturated humidity. Once the cells reached 80% confluence, they were treated as per the respective groups and further incubated for 24, 48, and 72 h. Subsequently, the original culture medium was discarded, and 100 μl of fresh medium containing 10% CCK-8 was added to each well. The plates were incubated for 1 h, and the optical density (OD) was measured at 450 nm using a microplate reader. The OD values were recorded at 24, 48, and 72 h to assess cell viability.

#### Colony formation assay to detect cell proliferation

Cells from the experimental and control groups were seeded at a density of 1.0 × 10^3^ cells/well in 6-well plates. After 2 weeks of incubation, the cultures were terminated, and the cells were fixed with paraformaldehyde and stained with crystal violet. The colonies were then counted.

#### Wound healing assay to detect cell migration

Cells in the logarithmic growth phase were trypsinized, centrifuged, resuspended, and counted. They were seeded into 6-well plates at a density of 6.0 × 10^5^ cells/well. The next day, when the cells reached a confluence of approximately 95%, a wound was created in the center of each well using a 20 μl pipette tip. The wells were washed with PBS and fresh culture medium was added. Images were captured at 0 h and again after 24 h of incubation using an inverted optical microscope. Cell migration was quantified using ImageJ software.

#### Flow cytometry to detect apoptosis rate

Cells were trypsinized, washed three times with PBS, and resuspended in 100 μl of 1 × binding buffer to a concentration of 1 × 10^6^ cells/ml. Five microliters of Annexin-V/FITC was added and the cells were incubated at room temperature for 15 min. Subsequently, 5 μl of propidium iodide was added, and the cells were incubated in the dark at room temperature for another 15 min. Four hundred microliters of 1 × binding buffer was added, and the cells were analyzed by flow cytometry.

#### Western blot analysis to detect protein expression

Cells were lysed using RIPA buffer to extract the protein. The protein was mixed with sample buffer and denatured at 100 °C for 10 min. The denatured protein was then separated by SDS-PAGE and transferred onto a PVDF membrane. After blocking the membrane, it was incubated with primary antibodies against Cleaved-caspase3 (diluted 1:2000), SIRT6 (diluted 1:1000), and GAPDH (diluted 1:1500) for 2 h at room temperature. This was followed by incubation with a secondary antibody diluted 1:2000 for 2 h at room temperature. Chemiluminescent substrate was added for visualization, and images were captured using a Bio-Rad chemiluminescent imaging system.

#### Dual-Luciferase reporter assay

The wild-type (WT) SIRT6 sequence containing the miR-34a-5p binding site and the mutant (MUT) SIRT6 sequence lacking the miR-34a-5p binding site were cloned into the pmirGLO reporter plasmid, yielding the recombinant vectors WT-SIRT6 and MUT-SIRT6, respectively. These recombinant vectors were then co-transfected with miR-34a-5p mimic or miR-34a-5p negative control (NC) into 293 T cells. The luciferase activity of the transfected cells was assayed using a dual luciferase reporter assay kit after 48 h of transfection.

### Statistical analysis

Statistical analysis was performed using SPSS 27.0 software. Each sample was assayed in triplicate with three replicate wells. The experiments were repeated three times, and the data were presented as mean ± SD. Independent samples t-test was used to analyze the differences between two groups. Pearson’s correlation analysis was employed to determine the expression relationship between miR-34a-5p and SIRT6 in cSCC tissues. A p-value of less than 0.05 (*) was considered statistically significant.

## Results

### MiR-34a-5p and SIRT6 expression in cSCC tissues and cell lines

**Fig. 1:**
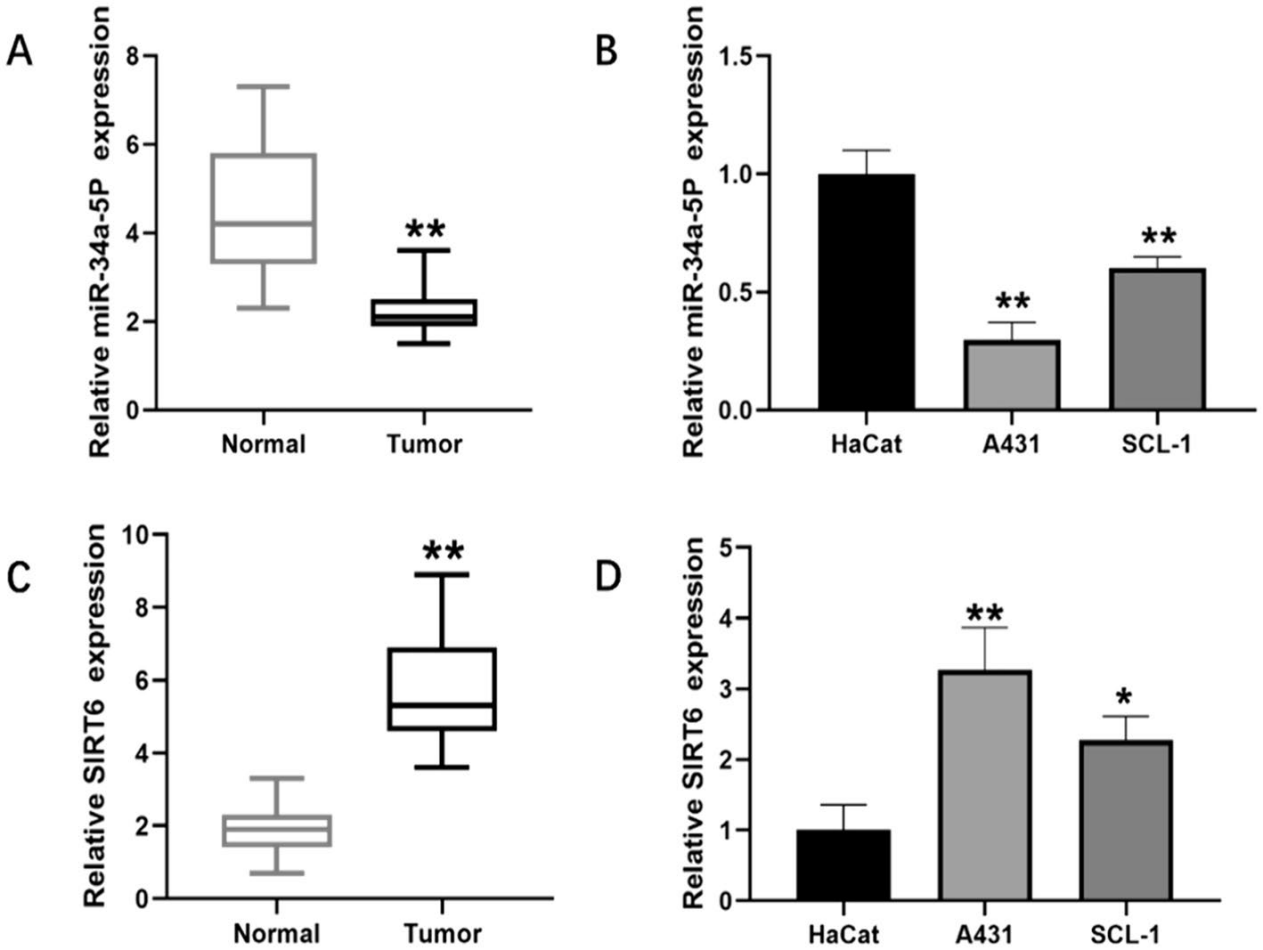
Downregulation of miR-34a-5p and upregulation of SIRT6 in cSCC. **A** RT-qPCR was used to assess the expression of miR-34a-5p in 15 cSCC tissue samples relative to normal skin tissue samples. **B** RT-qPCR analysis of miR-34a-5p expression in A431 and SCL-1 cells compared to HaCat cells. **C** RT-qPCR was used to evaluate the expression of SIRT6 in 15 cSCC tissue samples relative to normal skin tissue samples. **D** RT-qPCR analysis of SIRT6 expression in A431 and SCL-1 cells compared to HaCat cells. (*p < 0.05, **p < 0.01)

To assess the potential of miR-34a-5p and SIRT6 as biomarkers for the diagnosis of cSCC, we conducted qRT-PCR analysis to examine their expression levels in 15 cSCC biopsies and cultured cells. The control group consisted of normal skin tissue samples and HaCat cells. Compared to the control group, miR-34a-5p expression was significantly downregulated in both cSCC tissue samples and cells (Fig. 1A and B). Conversely, SIRT6 mRNA expression was increased remarkedly (Fig. 1C and D). These findings suggest the potential diagnostic value of miR-34a-5p and SIRT6 in cSCC.

### Correlation between miR-34a-5p and SIRT6 mRNA expression in cSCC tissues

**Fig. 2:**
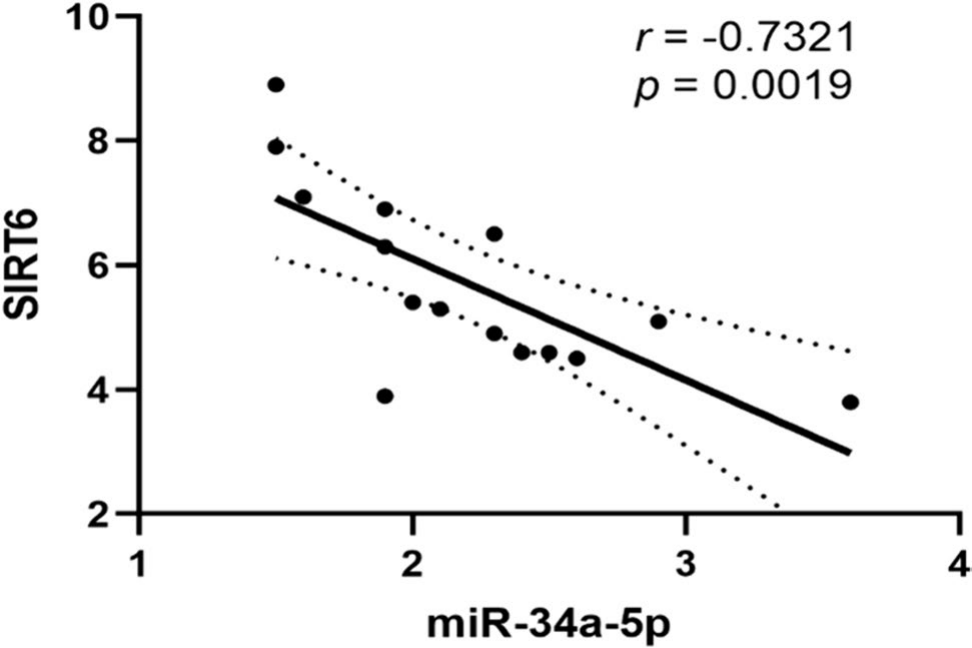
The expression of miR-34a-5p and SIRT6 mNRA in cSCC was negatively correlated

To further investigate the relationship between miR-34a-5p and SIRT6 in cSCC, we performed a Pearson correlation analysis. As shown in Fig. 2, SIRT6 mRNA expression was negatively correlated to miR-34a-5p in cSCC tissues (r = − 0.7321, p = 0.0019), suggesting that SIRT6 might be a target gene of miR-34a-5p.

### Effects of miR-34a-5p on cell growth, migration and apoptosis

**Fig. 3:**
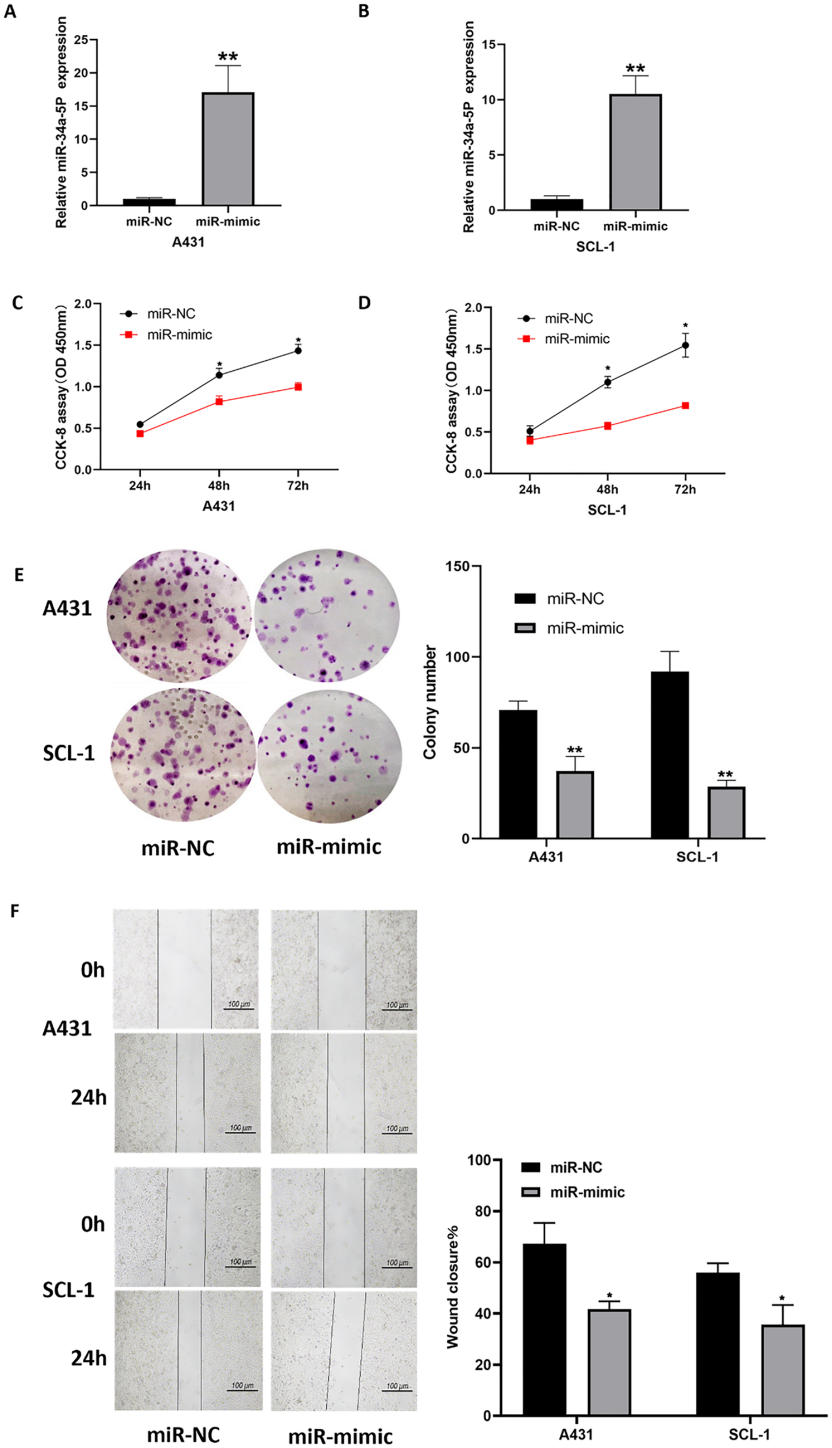

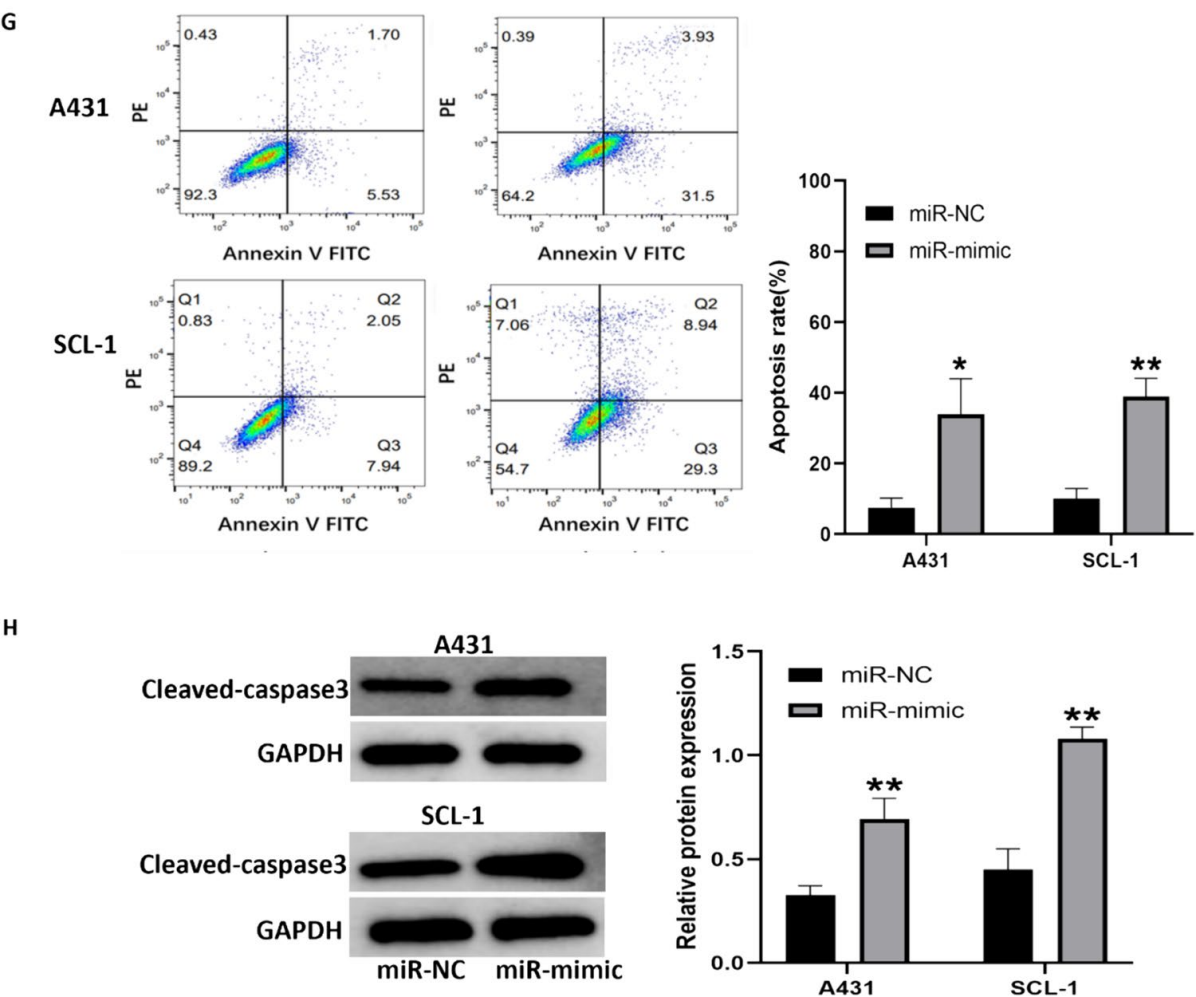
Inhibition of cSCC cell growth by MiR-34a-5p. **A**, **B** Relative expression of miR-34a-5p in A431 and SCL-1 cells transfected with NC mimic or miR-34a-5p mimic. **C**, **D** Cell counting kit-8 (CCK-8) viability assay performed at 24, 48, and 72 h post-transfection of miR-34a-5p mimic or NC mimic in A431 and SCL-1 cells. **E** Colony formation assay in A431 and SCL-1 cells transfected with NC mimic or miR-34a-5p mimic. Left panel: representative images; right panel: quantitative analysis. **F** Wound healing assay in A431 and SCL-1 cells treated with equal amounts of miR-34a-5p mimic or NC mimic. Left panel: representative images; right panel: quantitative analysis of wound closure. **G** Apoptosis detection by flow cytometry in A431 and SCL-1 cells transfected with NC mimic or miR-34a-5p mimic. Left panel: representative images; right panel: quantitative analysis. **H** Western blot analysis of apoptosis-related protein cleaved-caspase 3 expression levels in A431 and SCL-1 cells transfected with NC mimic or miR-34a-5p mimic. Left panel: representative images; right panel: quantitative analysis. (*p < 0.05, **p < 0.01)

To explore the role of miR-34a-5p in cSCC, miR-34a-5p mimics and negative controls were transfected into A431 and SCL-1 cells. The successful overexpression of miR-34a-5p following mimics transfection was confirmed by qRT-PCR (Fig. 3A and B). Subsequently, we utilized the CCK-8 assay to evaluate the effect of elevated miR-34a-5p levels on cell viability. Notably, up-regulation of miR-34a-5p significantly inhibited the proliferation of cSCC cells (Fig. 3C and D). Consistent with this, results from the clonogenic assay revealed a similar effect (Fig. 3E). Additionally, a dramatic reduction in migration ability by miR-34a-5p overexpression was also observed in both A431 and SCL-1 cells (Fig. 3F). Moreover, flow cytometry results showed that miR-34-5p greatly contributed to apoptosis of A431 and SCL-1 cells (Fig. 3G). To validate this, we further analyze the apoptosis-related protein levels. Compared to the control groups, cleaved-caspase 3 was increased in both A431 and SCL-1 cells transfected with mimics (Fig. 3H). Collectively, these results indicate that miR-34a-5p suppresses the progression of cSCC cells.

### SIRT6 is a target gene of miR-34a-5p

**Fig. 4:**
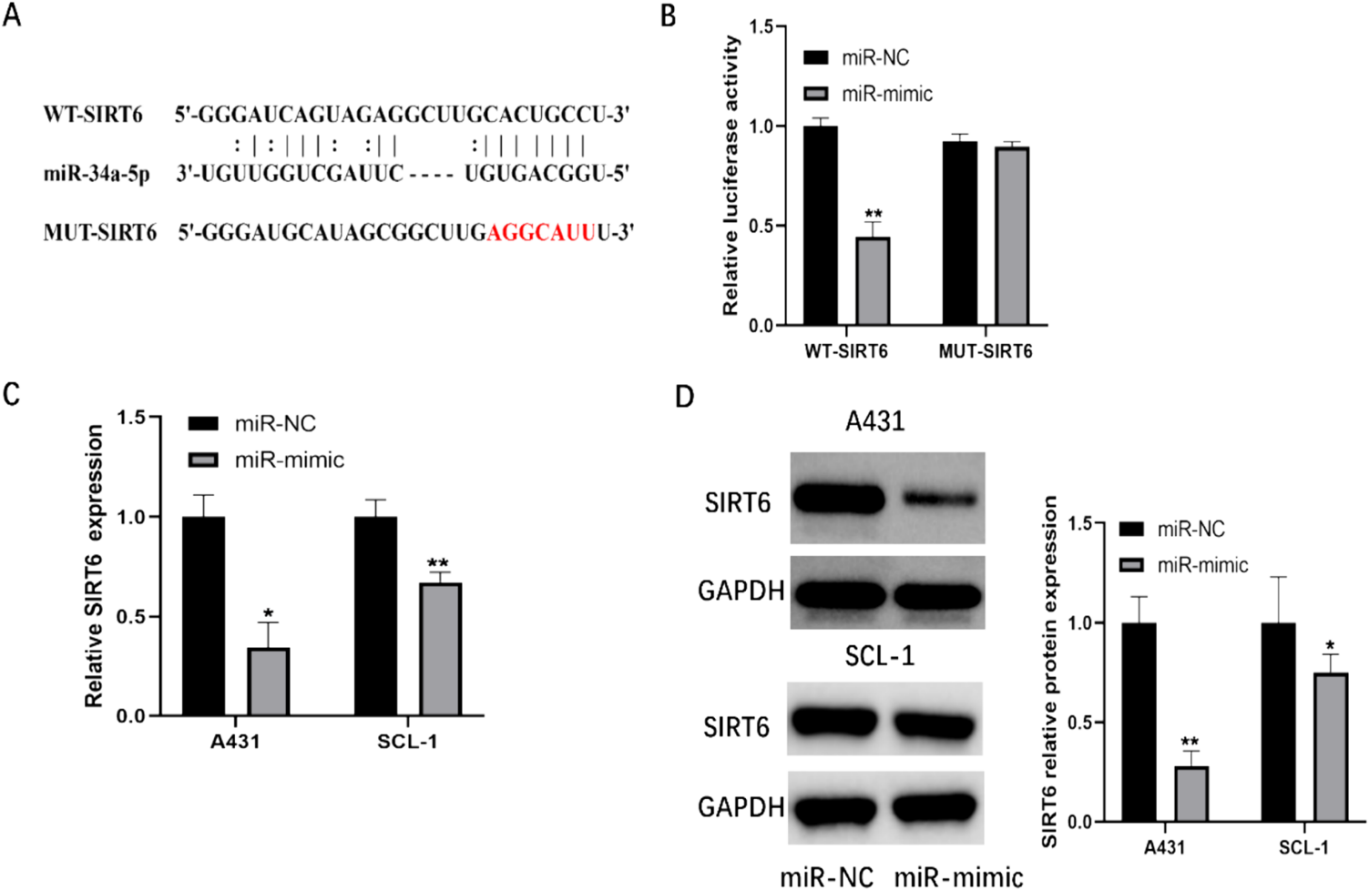
miR-34a-5p targets SIRT6 for regulation. **A** Representation of the putative binding site for miR-34a-5p in the wild-type and mutant SIRT6 3′UTR. **B** Luciferase reporter gene assay performed after transfection of luciferase vectors with miR-34a-5p mimic or NC mimic. **C** Relative expression of SIRT6 mRNA in A431 and SCL-1 cells transfected with NC mimic or miR-34a-5p mimic. **D** Relative expression levels of SIRT6 protein in A431 and SCL-1 cells transfected with NC mimic or miR-34a-5p mimic. Left panel: representative images; right panel: quantitative analysis. (*p < 0.05, **p < 0.01)

As shown in Fig. 4A, miR-34a-5p was predicted to target the 3′UTR of SIRT6 using TargetScan (www.targetscan.org). Given SIRT6’s involvement in pivotal pathological processes across various cancer types, it was selected as a putative target gene for miR-34a-5p.To verify whether SIRT6 is indeed a target of miR-34a-5p, we synthesized the 3′UTR of SIRT6 containing the possible miR-34a-5p binding sites with (wide-type) or without (mutant) mutations and inserted them into luciferase reporter vectors. After co-transfecting wild-type SIRT6 3′UTR or mutant SIRT6 3′UTR along with miR-34a-5p mimics or negative controls into 293 T cells, a luciferase reporter gene assay was performed. As shown in Fig. 4B, miR-34a-5p mimics significantly decrease the luciferase activity in 293 T cells transfected with the wild-type SIRT6 3′UTR, but did not affect those transfected with the mutant SIRT6 3′UTR, suggesting SIRT6 was a downstream target of miR-34a-5p. Furthermore, we anticipated that miR-34a-5p binding to the SIRT6 3′UTR would lead to a functional inhibition of SIRT6 expression. To confirm this, we transfected A431 and SCL-1 cells with miR-34a-5p mimics. As expected, both mRNA and protein levels of SIRT6 were down-regulated in A431 and SCL-1 cells by miR-34a-5p overexpression (Fig. 4C, D). In summary, our results demonstrated that miR-34a-5p directly target SIRT6 mRNA in cSCC cells.

### SIRT6 restoration rescues miR-34a-5p-mediated inhibition in cSCC cells

**Fig. 5:**
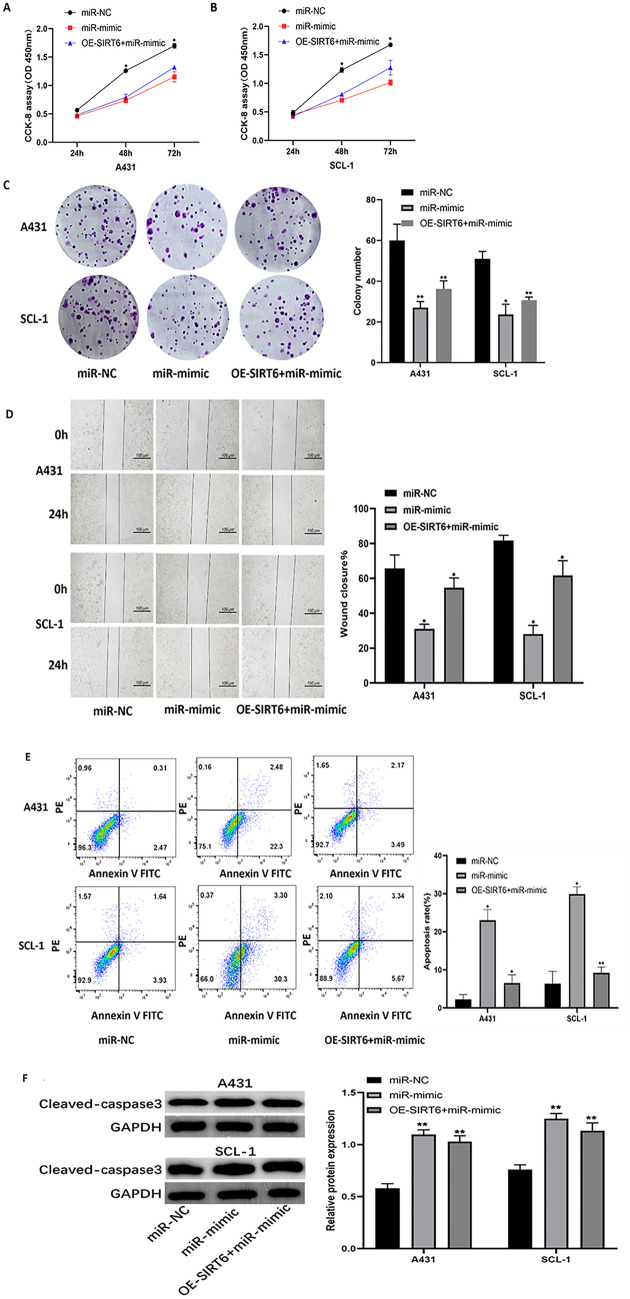
Overexpression of SIRT6 salvaged miR-34a-5p mediated inhibition of proliferation, migration and promotion of apoptosis in cSCC cells. **A**, **B** Cell counting kit 8 (CCK-8) was used to measure A431 and SCL-1 transfected with NC mimics, miR-34a-5p mimics, and miR-34a-5p mimics plus SIRT6 overexpression plasmid (OE-SIRT6 + miR-mimic) cell proliferation in cells at 24, 48 and 72 h time points; **C** clonal formation assay in A431 and SCL-1 cells transfected with NC mimics, miR-34a-5p mimics, and miR-34a-5p mimics plus SIRT6-overexpressing plasmids (OE-SIRT6 + miR-mimic), left: representative image, right: quantification of the number of clones formed; **D** scratch wound healing test in A431 and SCL-1 cells transfected with NC mimics, miR-34a-5p mimics, and miR-34a-5p mimics plus SIRT6 overexpression plasmid (OE-SIRT6 + miR-mimic), left: representative image, right: quantitative analysis of wound closure. **E** flow cytometry was used to analyze apoptosis in A431 and SCL-1 cells transfected with NC mimics, miR-34a-5p mimics, and miR-34a-5p mimics plus SIRT6-overexpressed plasmids (OE-SIRT6 + miR-mimic), left: representative image, right: quantitative analysis of apoptosis rate; **F** western blot transfected NC mimics, miR-34a-5p mimics, and miR-34a-5p mimics with SIRT6 overexpression plasmid (OE-SIRT6 + miR-mimic) expression levels of apoptosis-associated proteins were analyzed in A431 and SCL-1 cells, Cleaved-caspase3 (left: representative image, right: quantitative analysis). (miR-mimic group and OE-SIRT6 + miR-mimic group were compared with miR-NC group, respectively,*p < 0.05, **p < 0.01)

To further investigate whether miR-34a-5p exerted antitumor effects in cSCC cells by targeting SIRT6, we co-transfected miR-34a-5p mimics along with SIRT6 overexpression plasmids into A431 and SCL-1 cells. As anticipated, SIRT6 overexpression diminished the inhibitory effect of miR-34a-5p mimics on proliferation and migration of cSCC cells significantly (Fig. 5A–D). To gain further insight, flow cytometry experiments were conducted to determine whether SIRT6 overexpression could counteract the promotional effect of miR-34a-5p on cell apoptosis as well. Unsurprisingly, A431 and SCL-1 cells co-transfected with miR-34a-5p mimics and SIRT6 overexpression plasmids displayed significantly reduced cell apoptosis rates compared to cells transfected solely with miR-34a-5p mimics (Fig. 5E). Consistently, remarkable changes of cleaved-caspase 3 protein levels were also observed in cSCC cells co-transfected with SIRT6 and miR-34a-5p mimics compared to mi miR-34a-5p mimics-only treated cells. Taken together, these results suggested that SIRT6 restoration rescued the miR-34a-5p-mediated inhibitory effects in cSCC cells.

## Discussion

cSCC is a cutaneous malignancy that arises from keratinocytes within the epidermis and its appendages such as hair follicles and sebaceous glands. Currently, effective treatment is lacking and surgical resection remains the primary treatment modality [9]. However, given its predilection for exposed skin areas, surgical resection often lead to substantial tissue defects, causing both physical and psychological harm to patients. Besides, approximately 14–15% of cSCC cases manifest subclinical invasion, resulting in post-surgical recurrence, metastasis, and poor prognosis [10]. Hence, understanding the underlying mechanism of the development and progression of cSCC is crucial for novel effective therapeutic strategies.

MicroRNAs (miRNAs) is involved in many biological processes such as proliferation, senescence, and apoptosis. They also serve as vital regulators in tumor progression, metastasis, angiogenesis, and drug resistance development [6]. Notably, recent evidence showed that miR-34a-5p was associated with the pathogenesis of liver, breast, colorectal, and bladder cancers by targeting P53 [8, 11]. In addition, miR-34a-5p has been found to modulate cancer cell proliferation and invasion through regulating E2F3 and surviving expression [12]. In agreement with previous studies, we found that miR-34a-5p was down-regulated in cSCC. Notably, overexpression of miR-34a-5p significantly inhibited tumor cell proliferation and migration while promoting apoptosis, suggesting its tumor-suppressive role in cSCC. Next, SIRT6 was identified as a potential target gene of miR-34a-5p by Targetscan prediction. This interaction was further validated via a dual-luciferase reporter assay. Quantitative real-time PCR (qRT-PCR) and Western blot analysis confirmed the negative regulation of SIRT6 expression by miR-34a-5p, establishing a direct targeting relationship between the two. SIRT6 exhibits dual roles as a tumor suppressor or oncogene, depending on the specific cancer tissue or cell type [13]. While reduced SIRT6 expression was observed in colon, liver, pancreatic, and ovarian cancers, where it functioned as a tumor suppressor, elevated SIRT6 expression was demonstrated in prostate cancer, acute myeloid leukemia, multiple myeloma, and osteosarcoma [14–21]. These previous findings indicate that SIRT6 is closely related to the development of tumor. Similarly, our current study revealed high SIRT6 expression in cSCC tissues and cells. Furthermore, inhibition of SIRT6 expression by miR-34a-5p could attenuate cSCC progression, suggesting a potential oncogenic role of SIRT6 in cSCC.

In summary, our findings showed a down-regulated miR-34a-5p expression and up-regulated SIRT6 expression in cSCC cells and tissues, with miR-34a-5p suppressing cSCC progression by targeting SIRT6. These finding might provide novel insights into the pathogenesis of cSCC and highlight the importance of further elucidating the miR-34a-5p/SIRT6 pathway in cSCC tumorigenesis and development.

## Data Availability

The data that support the findings of this study are available on request from the corresponding author, [initials], upon reasonable request.
